# Longitudinal and Age-Related Implications of Primary and Secondary Control for Happiness

**DOI:** 10.1093/geroni/igab046.977

**Published:** 2021-12-17

**Authors:** Masahiro Toyama

**Affiliations:** University of the Ozarks, Clarksville, Arkansas, United States

## Abstract

While previous research addressed two distinct types of happiness, including hedonia and eudaimonia, the longitudinal associations of primary and secondary control with these happiness constructs had not been fully studied. The present study aimed to contribute to the literature by examining these associations and their age differences. Using data from the second and third waves of the Midlife in the United States (MIDUS; N = 4,963, aged 28 to 84 at baseline), the present study conducted structural equation modeling analyses to examine whether primary and secondary control predicted residualized changes over around a decade in the latent constructs of hedonia and eudaimonia and whether there were age differences in these associations. The results indicate that while only primary control predicted change in eudaimonia overall, the associations of primary and secondary control with changes in hedonia and eudaimonia differed by age. Particularly, in comparing these effects for younger and older individuals, primary control predicted increases in eudaimonia only for younger individuals, whereas secondary control predicted decreases in hedonia for younger individuals but predicted increases in eudaimonia for older individuals. Considering these findings, the importance of primary and secondary control for happiness may vary between adults of different ages, which is possibly due to their life priorities that may change with age. The present study suggested potential directions of future research further examining the role of primary and secondary control for happiness and exploring potential interventions to promote happiness, for example, by modifying primary and/or secondary control for adults of different ages.

